# Work-related psychosocial events as triggers of sick leave - results from a Swedish case-crossover study

**DOI:** 10.1186/1471-2458-11-175

**Published:** 2011-03-23

**Authors:** Hanna Hultin, Johan Hallqvist, Kristina Alexanderson, Gun Johansson, Christina Lindholm, Ingvar Lundberg, Jette Möller

**Affiliations:** 1 Karolinska Institutet, Department of Public Health Sciences, Division of Public Health Epidemiology, Norrbacka 7thfloor, SE-171 76 Stockholm, Sweden; 2 Uppsala University, Department of Public Health and Caring Sciences, Division of Preventive Medicine, Box 564, SE-751 22 Uppsala, Sweden; 3 Karolinska Institutet, Department of Clinical Neuroscience, Division of Insurance Medicine, SE-171 77 Stockholm, Sweden; 4 Linköping University, National Centre for Work and Rehabilitation, Department of Medicine and Health Sciences, IMH/RAR, SE-581 83 Linköping, Sweden; 5 Uppsala University, Department of Medical Sciences, Division of Occupational and Environmental Medicine, Uppsala University Hospital, SE-751 85, Uppsala, Sweden

## Abstract

**Background:**

Although illness is an important cause of sick leave, it has also been suggested that non-medical risk factors may influence this association. If such factors impact on the period of decision making, they should be considered as triggers. Yet, there is no empirical support available.

The aim was to investigate whether recent exposure to work-related psychosocial events can trigger the decision to report sick when ill.

**Methods:**

A case-crossover design was applied to 546 sick-leave spells, extracted from a Swedish cohort of 1 430 employees with a 3-12 month follow-up of new sick-leave spells. Exposure in a case period corresponding to an induction period of one or two days was compared with exposure during control periods sampled from workdays during a two-week period prior to sick leave for the same individual. This was done according to the matched-pair interval and the usual frequency approaches. Results are presented as odds ratios (OR) with 95% confidence intervals (CI).

**Results:**

Most sick-leave spells happened in relation to acute, minor illnesses that substantially reduced work ability. The risk of taking sick leave was increased when individuals had recently been exposed to problems in their relationship with a superior (OR 3.63; CI 1.44-9.14) or colleagues (OR 4.68; CI 1.43-15.29). Individuals were also more inclined to report sick on days when they expected a very stressful work situation than on a day when they were not under such stress (OR 2.27; CI 1.40-3.70).

**Conclusions:**

Exposure to problems in workplace relationships or a stressful work situation seems to be able to trigger reporting sick. Psychosocial work-environmental factors appear to have a short-term effect on individuals when deciding to report sick.

## Background

Sick leave entails a decision-making process, in which the sick individual either reports sick or goes to work despite some ill health. It has been suggested that non-medical factors in the work environment or related to private circumstances may influence the individual positively or negatively towards either decision and that work-related psychosocial events may act as absence incentives in this process [1-6]. It has also been suggested that sick leave is used as a coping strategy, allowing individuals to maintain and restore their health [4,7].

Several previous studies have shown that work-related psychosocial factors influence the risk of sick leave [8-17]. One possible interpretation of such associations is that an adverse psychosocial work environment increases the risk of illness which in turn leads to sick leave [8-18]. But other mechanisms have been discussed [9,10,13-16,18]. One such mechanism is that these factors affect the risk of reporting sick when one is ill [1-4,10,18].

Several of the non-medical factors consciously, or non-consciously, influencing the decision to report sick may operate in a short period before the decision-making. Thus, the induction period before the effect becomes manifest is short and such factors can, therefore, be viewed as triggers [3]. In the last decade, the case-crossover design has been introduced as an effective method to study potential triggers [19,20]. The study design implies that the cases contribute with both case and control information. Initially, the design was used to study triggers of myocardial infarction [20,21], but it has also been used to study non-medical risk factors in decision-making processes, for instance, triggers of making the decision to contact a general practitioner [22,23]. However, so far it has not been used in studies of sick leave.

The aim of this study was to investigate whether work-related psychosocial events can trigger the decision to report sick when ill. Our focus is on non-medical factors interacting with illness and not whether psychosocial events may increase the risk of reporting sick when not ill.

## Methods

### Study design

We used data from the TUFS project (an acronym in Swedish for "Triggers of sick leave"), which was designed as a case-crossover study nested within a cohort, and carried out at six geographically disparate Swedish workplaces, between April 2005 and February 2007.

In a case-crossover study, cases act as their own controls. Exposure frequency during a time period in close proximity to the outcome, the case period, is compared with exposure frequencies during one or more control time periods for the same individual, and if an exposure has a trigger effect, it should be more frequent in time periods proximate to the outcome than in more distant time periods [19,20].

### Study sample

The six workplaces were selected to cover three occupational sectors; manufacturing (one manufacturing plant), health care (four public and municipal healthcare facilities), and office work (one insurance company). The project group approached the workplaces through the executive management and the project was approved by workplace union representatives and by Stockholm's Regional Ethics Committee.

The workplaces employed between 30 and 1 200 individuals. Human resource staff at the workplaces identified all employees who were not on parental leave, currently on sick leave for more than 30 days, or on other leave of absence. In total, 3 149 individuals were found and after detailed corrections on eligibility criteria, 3 020 were considered eligible for participation in the cohort. The study cohort consists of the 1 430 individuals (47%) who returned a baseline postal questionnaire and a consent form, sent to their home addresses.

### Data sources

We used three sources of data: 1) baseline questionnaire data regarding health, private life, and work environment, 2) daily reports from the workplaces on start and end dates of all new sick-leave spells among the participants during a follow-up, which varied from 3 to12 months between the different workplaces, and 3) telephone interview data, concerning exposure to potential trigger factors, collected from the participants during the first days on sick leave.

New sick-leave spells were reported daily by e-mail or fax from the employers. The logistics for this was part of an already existing organisation for staff management at five of the six workplaces; and at one, a specific group of informants were recruited. At one workplace, daily reporting of sickness absence was not possible, and instead reporting was carried out on a weekly basis. Group-level data on sick-leave spells among non-participants was also reported to the project.

In the telephone interview, exposure to work-related psychosocial events was ascertained and recorded in an interview form with set questions and answer categories. The selection of exposures was based on the theoretical concepts of the Illness Flexibility Model, formulated by Johansson and Lundberg [1,2], and were further specified in a focus group study at the design phase of the project.

### The sick-leave spells

A sick-leave spell was defined as each time an employee reported sick to their workplace. All new spells from participants that were reported during the follow-up, regardless of length and grade, were included, except planned sick leave (i.e., for planned surgery). Since the unit of analysis was sick-leave spells, some participants contributed with more than one spell during the follow-up. However, except during an initial pilot period, individuals that had participated in three interviews were not contacted if on sick leave again (45 spells were considered non-eligible for this reason), leaving a total of 877 sick-leave spells eligible for inclusion in the study. In 679 of these spells an interview was conducted, and in 198 (23%) spells the absentee declined or could not be reached. In 111 (13%) of the interviewed spells, the absentee did not have time or strength to complete a full interview, and a shortened version of the interview, with no exposure information, was conducted. In 22 of the spells (3%), more than 14 days had passed before the interview, and they were excluded at analysis. The total number of sick-leave spells included in the analyses was thus 546 (62% of all recorded eligible sick-leave spells).

### Exposures

Nine hypothetical non-medical work-related triggers were measured in the interviews. Four exposures concerned the respondent's relationship with his or her superiors (exposure to "insufficient appreciation from superior", "quarrel or conflict with superior", "criticism from superior", and "disregarded or brushed aside by superior"). These were analysed separately and combined into one measure called "problems in the relationship with superior". Respondents were considered as exposed if exposure was reported in at least one of the separate items.

Two exposures measured the relationship with colleagues ("insufficient appreciation from colleagues" and "quarrel or conflict with colleagues"). These were also analysed separately and combined into a measure called "problems in the relationship with colleagues" in the same way as "problems in the relationship with superior".

The final three exposures concerned experiencing "discrimination, bullying, sexual harassment, or other type of harassment", "unpleasant or uncomfortable tasks, tasks for which you are not skilled enough or which you, for other reasons, wish you could get out of performing", and "a very stressful work situation, indicated by more tasks, fewer staff or a larger field of responsibility than usual".

Each exposure was ascertained through a set of questions, following a similar sequence: First respondents were asked whether they had been exposed at any time during the last year. If so, exposure was assessed on each workday during a two-week period prior to the first sick-leave day and during the first sick-leave day.

**Figure 1:**
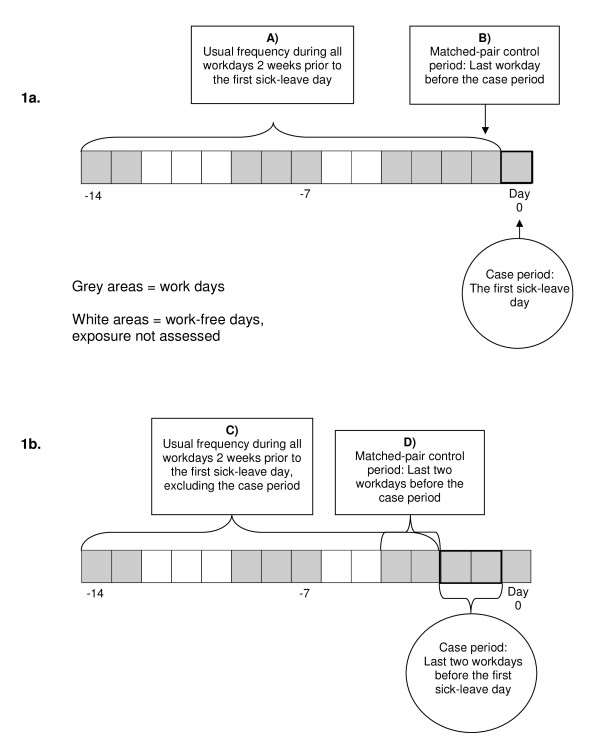
**Schematic picture of the time periods used in the analyses**. 1a shows the case and control periods used for the exposures "unpleasant tasks" and "very stressful work situation". 1b shows the case and control periods used for the exposures "problems in relationships with superior", "problems in the relationship with colleagues" and "discriminated, bullied or sexually harassed".

We used two types of control information. The usual frequency of exposure was based on all exposed work days during the two-week period before the first sick-leave day, excluding the chosen case period (Figure 1). The matched-pair control period had the same length as the case period, and was chosen from the same two-week period (Figure 1).

For the exposures regarding unpleasant tasks and a very stressful work situation, the case period was defined as the first sick-leave day (Figure 1a) and the matched-pair control period consisted of the last workday before the first sick-leave day. For problems in the relationships with colleagues and with superiors, and discrimination, bullying, or harassment, the case period was defined as the last two workdays before the first sick-leave day (Figure 1b) and the matched-pair control interval consisted of the last two workdays before that period.

### Background factors

The following variables were extracted from the baseline questionnaire for descriptive analyses: age, sex, having a partner, having children younger than 18 years, and percentage of housework performed. Self-rated health was measured by a question based on the first item from the Swedish version of SF36 [24], however slightly modified. Adjustment latitude and attendance requirements are two concepts from the Illness Flexibility Model, and these were measured with questions used in previous studies of these factors (definitions of these are given in Table 1) [1]. Occupational titles from the questionnaire were coded into the 2 digit SEI codes (Socioeconomic Classification) of Statistics Sweden [25].

**Table 1:** Characteristics of the interviewed individuals (n = 432)

Individual baseline variables		%
**Age**	Mean: 43 years	

**Sex**	Women	60%

**Occupational sector**	Health care	23%
	Manufacturing	54%
	Office work	23%

**Socioeconomic status**	Unskilled manuals	24%
	Skilled manuals	19%
	Lower non-manuals	20%
	Middle non-manuals	30%
	Higher non-manuals	5%

**Self-rated health^1^**	Very good or good	78%
	Fair, bad or very bad	22%

**Housework^2^**	All (100%)	23%
	Half or more	57%
	Less than half	16%

**Partner relationship**	Yes	76%

**Have children (aged 0-18 years)**	Yes	44%

**Attendance requirements^3^**	Always or often	9%
	Sometimes	39%
	Seldom or never	49%

**Adjustment latitude^4^**	Never	28%
	Seldom	25%
	Sometimes	36%
	Often	9%

**Number of sick-leave days during the 12 months prior to inclusion**	None	18%
	1-7 days	51%
	8-30 days	23%
	31-90 days	2%
	More than 90 days	3%

1 Measured by the question: "What do you consider your health status as in general?".

2 Measured by the question: "How large a share of the total amount of house work do you perform?".

3 Measured by the question: "Can staying home for one or two days because of illness, be difficult for you because of work demands?".

4 Measured by the question: "If you are tired, out of sorts, or have a headache, are you able to adjust work to how you are feeling?".

When interviewed, the respondents were asked to state their reason for taking sick-leave. These self-reported health problems were coded into eight broad categories. Furthermore, the respondent was asked whether there were any circumstances (besides illness) that had influenced the respondent's decision to report sick. These answers were coded into "work related" and/or "private related". The respondents' work ability when reporting sick was measured with a question based on the first item of the Work Ability Index [26], however slightly modified.

### Statistical analyses

When applying the usual frequency approach (using the usual frequency of exposure during the two-week period), the odds ratios (OR) were calculated using a Mantel-Haenszel estimator with 95% confidence intervals (CI) for sparse data [27]. These reflect the ratio of the observed odds of exposure during the case period, to the expected odds of exposure.

In the matched-pair interval approach (using control information based on the matched-pair control period), we used conditional logistic regression, with each sick-leave spell being regarded as one stratum [20,27]. The OR reflects the odds of exposure in the case period compared to in the control period.

The odds ratios can be considered as estimates of the incidence rate ratio comparing exposed to unexposed conditions [28].

The interviewers used special codes to indicate when the respondent could not pinpoint which day he or she had been exposed. In all analyses of matched-pair control periods and in the analyses of usual frequency control periods for the exposures "discriminated, bullied or sexually harassed", "unpleasant tasks" and "very stressful work situation" these "uncertain exposures" were considered as exposed, but in the usual frequency analyses from the combined measures of "problems in the relationship with a superior" and "problems in the relationship with colleagues" they were considered as missing, since the unknown timing of the exposure event did not allow for the adding of the number of exposed days over several questions. Alternative analyses were made using stricter coding of such days. Other alternative analyses were performed to assess possible biases, including analyses excluding cases reporting more than one exposure in the case period, analyses differentiating between cases with and without illness symptoms prior to the first sick-leave day, and separate analyses of cases where the respondents had worked part of the first sick-leave day. An estimated sick-leave incidence rate was calculated, for participants and non-participants, with person-time based on calendar days, including both the days on sick leave and work-free days.

## Results

Among the participants, 39% had at least one recorded sick-leave spell during follow-up. The estimated sick-leave incidence rate for the entire 3-12 month follow-up was 2.85 spells/1 000 person days among participants compared to 4.30 spells/1 000 person days among non-participants. The 546 sick-leave spells that were included in the this study are derived from 432 individuals, which means that 79% of the interviews came from unique participants. Characteristics of these individuals are presented in Table 1. More than half of the self-reported health reasons for the sick-leave spells were colds, influenzas, or headaches (Table 2). The median self-reported work ability when reporting sick was 2 on a 0-10 scale, and it did not differ significantly between sick-leave spells exposed or unexposed to any of the exposures under study.

**Table 2:** Characteristics of the included sick-leave spells (n = 546)

Interview variables		
**Time from first sick-leave day until interview**	Median	2 days
	Min	0 days
	Max	14 days

**Exposure to work-related psychosocial events during the last year**	Insufficient appreciation from superior	31.3%
	Quarrel or conflict with superior	8.2%
	Brushed aside/disregarded by superior	17.0%
	Criticized by superior	14.3%
	Insufficient appreciation from colleagues	9.0%
	Quarrel or conflict with colleagues	12.8%
	Discriminated, bullied, sexually harassed, or harassed in other way	6.0%
	Unpleasant tasks, not skilled enough, wants to get out of	20.5%
	Very stressful work situation; unusually high workload, tight deadlines, more tasks, or larger field of responsibility	66.5%

**Self-reported health problem at sick leave **(possible to report more than one health problem)	Infections, colds, viruses, influenzas, unspecified fever etc.	48.2%
	Stomach or intestinal conditions	20.3%
	Muscle and/or joint pains or trauma	6.8%
	Back/neck pain	8.4%
	Stress/tiredness/low-spiritedness	7.1%
	Headache/migraine	5.7%
	Other/not specified	12.3%

**Self-estimated work ability when reporting sick^1^**	75^th ^percentile	4
	Median	2
	25^th ^percentile	0

**Self-reported reason for taking sick-leave in addition to the stated disorder**	None	78.2%
	Private/home related	5.0%
	Work related	15.0%
	Both work and private related	1.8%

Table showing values of median, min and max and percentages.

1 Measured by the question: "If you imagine a scale where zero is equivalent to no work ability and ten to your best possible work ability, how many points would you give the work ability you had when you reported sick?"

Exposure to at least one work-related psychosocial event during the last year was reported in 81% of the sick-leave spells, and in 19% at least one exposure during the case periods was reported. The most reported exposure was a very stressful work situation.

A trigger effect was found for exposure to problems in the relationship with a superior during any of the last two workdays before the first sick-leave day, when using the usual frequency approach (OR 3.63; 95% CI 1.44-9.15) (Table 3). When using a matched-pair control period the OR was 2.33 but not statistically significant (CI: 0.60-9.02). A similar pattern was seen for problems in the relationship with colleagues, with a usual frequency OR of 4.68 (CI 1.43-15.30) and 2.50 (CI 0.49-12.89) when using the matched-pair control period. We performed stratified analyses for these exposures, separating cases that did and did not report illness symptoms before the first sick-leave day, and in both sub-groups ORs in the same direction were suggested, but the effect estimates from the spells with no symptoms prior to the first sick-leave day were not statistically significant (data not shown).

**Table 3:** Estimated odds ratios of sick leave on an exposed day relative to an unexposed day

Type of work-related psychosocial event	Case period	Control period	OR	95% CI
Problems in the relationship with a superior	Last two workdays before first sick-leave day	Two-week usual frequency	3.63	1.44-9.15
		Matched pair control period, last two workdays before case period^1^	2.33	0.60-9.02

Problems in the relationship with colleagues	Last two workdays before first sick-leave day	Two-week usual frequency	4.68	1.43-15.30
		Matched pair control period, last two workdays before case period^2^	2.50	0.49-12.89

Discriminated, bullied, sexually harassed, or harassed in other way	Last two workdays before first sick-leave day	Two-week usual frequency	4.00	0.31-52.03
		Matched pair control period, last two workdays before case period^3^	1.00	0.06-15.99

Unpleasant tasks, not skilled enough, wants to get out of	First sick-leave day	Two-week usual frequency	2.59	0.94-7.16
		Matched-pair control period, last workday before case period^4^	1.20	0.37-3.93

Very stressful work situation; unusually high workload, tight deadline, more tasks, or larger field of responsibility	First sick-leave day	Two-week usual frequency	2.27	1.40-3.70
		Matched-pair control period, last workday before case period^5^	2.70	1.31-5.58

Odds ratios (OR) and surrounding 95% confidence intervals are presented for the two different analytical approaches of control information.

1 There were 7 cases who reported problems in the relationship with a superior only during the last two workdays before sick leave, as compared to 3 cases who reported exposure only during the last two workdays before the case period. Twenty cases were exposed during both periods.

2 There were 5 cases who reported problems in the relationship with colleagues only during the last two workdays before sick leave, as compared to 2 cases who reported exposure only during the last two workdays before the case period. Six cases were exposed during both periods.

3 There was 1 case who reported being discriminated, bullied, sexually harassed or harassed in other way only during the two workdays before sick leave, and 1 case who reported exposure only during the last two workdays before the case period. One case reported exposure during both periods.

4 There were 6 cases who reported exposure to unpleasant tasks only during the first sick-leave day, as compared to 5 cases who reported exposure only during the last workday before sick leave. Ten cases were exposed during both periods.

5 There were 27 cases who reported exposure to a very stressful work situation only during the first sick-leave day, as compared to 10 cases who reported exposure only during the last workday before sick leave. Thirty-six cases were exposed during both periods.

Anticipating a very stressful work situation on the first sick-leave day, resulted in an OR of sick leave of 2.27 (95% CI 1.40-3.70) when using the usual frequency approach, and an OR of 2.70 (95% CI 1.31-5.58) when using a matched-pair control day (Table 3).

The potentially triggering effect of exposure to "discrimination, bullying, or sexual harassment" the two previous workdays, or expected exposure to "unpleasant tasks" on the first sick-leave day was non-significant.

## Discussion

We found an increased risk of sick leave after the respondents had been exposed to problems in their relationship with a superior or colleagues in the previous two workdays. Furthermore, respondents were more inclined to report sick on days when they expected a very stressful work situation, than on days when they did not.

The specificity of the outcome measure is high, since the individuals contributing with case information confirmed their sick-leave status at the interview. Decreased sensitivity is only a problem if associated with exposure. If, at all, cases exposed to work-related psychosocial events might not be reported, this would result in an underestimation of the effect estimates.

To minimize a differential influence of case status on the retrospective exposure information between the case and the control periods, both the participants and the interviewers were reinforced to treat all 14 days in the recall period equally by not informing them of the hypothetical length of the induction period. The results also showed the same tendency when using the usual frequency or the matched-pair type of control data, which supports the validity of the control information and argues against important recall bias. It should also be noted that the exposure information was collected as a parallel series of events and there was no explicit wording on how it might be related to sick leave. Instead the participants were asked in separate questions to give their view on different reasons and causes for their recent sick-leave spell. To minimize memory problems we tried to make the recall period as short as possible and the median time from the first day of sick leave to the interview was two days. Previous research also indicates that unpleasant events like our exposures are more correctly recalled than neutral ones [29]. In two additional analyses we coded uncertain exposure events as unexposed and as missing, respectively, but the effect estimates generated by those analyses were still of similar magnitude and direction as those reported in Table 3 (data not shown). Social desirability may influence how exposure is reported, especially for the case period, but it is difficult to know the direction of its influence. Our general impression from the specific questions on the participant's own view of the reasons for their sick leave is however, that they primarily stressed the illness and were not keen on mentioning anything else. If this tendency was operating in our collection of exposure information as well it would lead to an underestimation of the reported effects. Differential misclassification of exposure has been a primary methodological concern since the introduction of the case-crossover design [19], but later experience from more than 300 research reports and from specific methodological studies has proven that the methodology is rather robust in this respect [30].

For most cases, when measuring exposure to "very stressful work situation" and "unpleasant tasks", exposure in the case period measured expected exposure, if one had worked. The control periods were, on the other hand, assessed retrospectively regarding experienced workdays. It is hard to estimate the effect of the qualitative difference in information regarding experienced exposure in comparison to expected. However, 11% of the cases reported working during part of the first sick-leave day, which would make their case and control information more comparable. When separate analyses were made for these cases, an increased OR for exposure to a very stressful work situation was seen for them as well (data not shown).

An important element of case-crossover design and analyses is estimating the length of the hazard period, i.e. the period after a trigger begins when one expects to find an increased risk of the outcome. Based on the Illness Flexibility Model and other theories of sick leave from a coping perspective we expected the exposures related to work tasks to have an effect mainly if present on the first sick-leave day, which would indicate that the individual may use sick leave as a means to cope with these tasks [1,4,7]. On the other hand, it is likely that exposures concerning relationships with other individuals have to have happened to have an effect, and we therefore chose a case period of the last two workdays before the sick leave. The number of exposure events reported during these chosen case periods, as compared to the rest of the days in the two-week period also indicated that we had made the correct assumptions. Over or underestimation of the hazard period would both lead to an underestimation of the true effect.

One strength of the self-matching element of the case-crossover design is that all stable confounders, measured and unmeasured, are controlled for. However, confounding from other triggers may still occur [20]. We have not found any previous studies on triggers of sick leave; however, we acknowledge the possibility of co-variation between the different exposures in this study. Unfortunately, the statistical power constrains us from adjusting for such confounding and from exploring interaction effects. In 17% of the interviews, the respondents reported more than one exposure in the case period. Excluding double-exposed cases slightly mitigated the effect estimates, but did not change our conclusions.

Being ill, or experiencing symptoms of illness may make an individual more or less prone to, for instance, engage in conflicts with colleagues. Based on our study we cannot conclude which is more likely. Moreover, an ill individual may perceive situations as more or less problematic or stressful than when well, in which case the illness would confound the presented results. Both circumstances imply that the individual was ill or experienced symptoms when exposed to the psychosocial event. However, stratified analyses on cases with and without reported symptoms prior to the first sick-leave day both indicated ORs in the same direction, although the effect estimates from the cases without prior symptoms (24% of all cases) were not statistically significant (data not shown).

The overall participation in the cohort was 47%, which limits the possibility to generalize the results. Descriptive comparisons of participants and non-participants revealed that non-participation was more common in the healthcare sector, and among younger employees. The estimated sick-leave incidence rate was higher among non-participants than among participants. Of the recorded participant sick-leave spells, 38% were not interviewed. However, more than half of the individuals contributing to these excluded sick-leave spells were interviewed concerning other spells during follow-up. Descriptive comparisons between sick-leave spells included in analyses and sick-leave spells in which only a short interview was conducted, did not reveal any differences regarding self-reported reasons for reporting sick or self-rated work ability at the time of reporting sick. The main reasons for declining to be interviewed were lack of time or feeling too ill. However, if exposure was the true reason for either declining to participate completely in the study, or declining to be interviewed during a specific sick-leave spell, this could imply that we miss exposed cases, leading to underestimated ORs.

No previous study has investigated if work-related psychosocial events can trigger the decision to report sick. Our results indicate that expected exposure to very stressful work situations can trigger the decision to report sick when ill, in a way that could be regarded as the type of coping suggested by Kristensen [4,7]. In the Illness Flexibility Model, formulated by Johansson and Lundberg, the term work-related absence incentives is used to describe how work-related factors such as conflicts or a stressful work situation may push the individual towards the decision to report sick [1-3]. They suggest that many of the conditions in the model have a very short induction time, but the concept of absence incentives has never been empirically tested [3]. Our results fit their theoretical assumptions well. Previous studies have shown that high work demands, effort-reward imbalance, low supervisor support, and low management quality increase the risk of sick leave [8,10,12,14-17]. The effect of low social support from colleagues on sick leave is less explored [6,12,17]. Although several different mechanisms have been discussed, the most common interpretation of such associations so far has been that these factors cause ill health among employees, which in turn increases the risk of sick leave [8-10,12,14-17]. By using a case-crossover approach we showed that the specific psychosocial events, which such factors reflect, appears to have an additional immediate effect on the decision-making process of the ill individual. However, the number of cases which were exposed in the case period was low for several of the studied exposures, and the absolute effect of exposure may therefore be quite low. It is also important to note that there may still exist a long-term or cumulative effect of recurrent psychosocial events, which cannot be studied with a case-crossover design. Such an effect may modify the reported trigger effect.

In this study we cannot distinguish between the effect of psychosocial events in interplay with illness and the possible independent effect of psychosocial events. Studying the effect of psychosocial events independently of illness would require a different design, including a case series without illness. In Sweden as in other countries, a legal requirement for sickness benefit is reduced work ability for medical reasons [31,32]. The effect of non-medical trigger factors within the work environment can be assumed to be dependent on the level of work-ability reduction experienced by the individual. We consider a complete lack of work ability to be an absolute criterion for sick leave. Sick leave in case of full work ability means cheating. In between these two extremes the reduced work ability due to illness implies a relative indication for sick leave and the decision might also be influenced by non-medical circumstances, such as the triggers studied here. More than half of the self-reported health problems among the cases were related to colds, influenzas, or headaches, and the median self-estimated work ability when reporting sick was 2 on a 0-10 scale. How specific health problems translate into occupation-specific work-ability reduction is unclear [18]. The limited statistical power of this study impedes sub-group analyses of type of illness, degree of work-ability reduction, and general health status as well as more general effect modification analyses of stable risk indicators such as occupation, sex, and age. Such effect modification is plausible and should be investigated in future studies. Nevertheless, our results suggest that the work ability of some of the included cases must have been sufficiently large to allow for other factors to have an effect.

## Conclusions

This is the first study of the trigger effects of work-related psychosocial events on the decision to report sick when experiencing illness. This study needs to be repeated in samples that allow for broader generalization and for investigation of effect-modification. However, the results imply that psychosocial work-environmental factors appear to have a short-term effect on individuals' decision to report sick.

## Competing interests

The authors declare that they have no competing interests.

## Authors' contributions

All authors had full access to all of the data (including statistical reports and tables) in the study and can take responsibility for the integrity of the data and the accuracy of the data analysis. HH participated in the design and coordination of the study, collected data, performed the statistical analyses, contributed to the interpretation of the data, and drafted the manuscript. JH conceived the study idea, participated in its design and coordination, contributed to the interpretation of the data, and helped to draft the manuscript. KA participated in the design of the study, contributed to the interpretation of the data, and helped to draft the manuscript. GJ participated in the design of the study, contributed to the interpretation of the data, and helped to draft the manuscript. CL participated in the design of the study, contributed to the interpretation of the data, and helped to draft the manuscript. IL participated in the design of the study, contributed to the interpretation of the data, and helped to draft the manuscript. JM conceived the study, participated in its design and coordination, supervised HH in the statistical analyses, contributed to the interpretation of the data, and helped to draft the manuscript.

## Pre-publication history

The pre-publication history for this paper can be accessed here:

http://www.biomedcentral.com/1471-2458/11/175/prepub
